# Fraxinellone mitigates acute lung injury by targeting HIF-1α to suppress pyroptosis and inflammation

**DOI:** 10.3389/fimmu.2026.1826165

**Published:** 2026-07-16

**Authors:** Bing Zhang, Xiaoning Lu, Yuefei Zhang, Xinyu Shen, Zhongbing Tan, Junling Leng, Yifei Chen, Hui Shen, Yong Li

**Affiliations:** 1 Department of Emergency Medicine, Affiliated Hospital of Yangzhou University, Yangzhou, China; 2 Department of Cardiothoracic Surgery, Nanjing Medical University Affiliated Suqian First People’s Hospital, Suqian, China; 3 Department of Cardiovascular Medicine, Affiliated Hospital of Yangzhou University, Yangzhou, China; 4 Department of Critical Care Medicine, Affiliated Hospital of Yangzhou University, Yangzhou, China

**Keywords:** acute pulmonary contusion, fraxinellone, hypoxia-inducible factor-1α, pyroptosis, thrombin

## Abstract

**Purpose:**

Acute pulmonary contusion (APC), a life-threatening trauma-associated condition, currently lacks targeted pharmacological therapies. Fraxinellone (FRA) inhibits Hypoxia-inducible factor-1α (HIF-1α) synthesis, but its binding mechanism and anti-APC action remain unclear. This study was designed to assess the therapeutic potential of FRA, a natural substance with known anti-inflammatory properties, as a means of attenuating APC progression through the inhibition of HIF-1α.

**Methods:**

An *in vivo* APC model was generated via weight-drop lung injury in C57BL/6 mice, and an *in vitro* model was established using thrombin (THR)-injured MLE-12 cells. The efficacy of FRA (10 μM *in vitro*; 5 mg/kg *in vivo*) was evaluated through cell viability assays, measurement of inflammatory cytokines via enzyme-linked immunosorbent assay (ELISA), histopathological examination, and molecular analyses including Western blot (WB) and real-time quantitative polymerase chain reaction (RT-qPCR). Mechanistic validation was performed through HIF-1α siRNA knockdown and mutation, ML228-mediated activation, and molecular docking simulations.

**Results:**

FRA notably diminished APC severity by reducing pulmonary edema (wet/dry ratio), lowering pro-inflammatory cytokine levels ((interleukin-1β [IL-1β], interleukin-6 [IL-6], interleukin-18 [IL-18]), and decreasing pyroptosis markers (NOD-like receptor family pyrin domain containing 3 [NLRP3], gasdermin D [GSDMD]) in both *in vitro* and *in vivo* models. Molecular docking revealed FRA binding to HIF-1α at Arg258, and HIF-1α activation abrogated the protective effects of FRA. HIF-1α knockdown and mutation reproduced the anti-pyroptotic effects of FRA, confirming pathway specificity.

**Conclusion:**

FRA mitigates APC progression by targeting HIF-1α to suppress NLRP3/GSDMD-mediated pyroptosis and inflammation, providing a novel therapeutic approach for trauma-induced lung injury.

## Introduction

Acute pulmonary contusion (APC), a common outcome of direct or indirect thoracic trauma (≈50%), is a form of life-threatening pulmonary injury that often requires specialized hospitalization for clinical management (1). As a significant factor in mortality within critical care environments, APC can develop into acute respiratory distress syndrome (ARDS) in severe instances. Despite its clinical importance, the molecular mechanisms underlying APC remain poorly defined, and current therapeutic strategies are limited to supportive care, such as protective mechanical ventilation, with no effective targeted pharmacological interventions (2–4). This knowledge gap underscores the urgent need to clarify the molecular drivers of APC and to develop effective pharmacotherapies.

Mechanical trauma from vehicular accidents or falls leads to structural damage of the lung parenchyma in severe APC cases. Secondary inflammatory cascades further disrupt the alveolar-capillary barrier, drive pulmonary edema, and exacerbate systemic hypoxia (5–7). Hypoxia, a hallmark of APC pathophysiology, activates hypoxia-inducible factors (HIFs) through oxygen-sensing pathways (8). Evidence has increasingly implicated hypoxia-inducible factor-1α (HIF-1α) in regulating innate immunity under hypoxic conditions, particularly via synergistic interactions with nuclear factor-κB (NF-κB) (9, 10), suggesting that HIF-1α serves as a master regulator of inflammatory responses in alveolar epithelial cells (AECs) in the context of hypoxia. AECs are the primary cells that are damaged in APC, which leads to adverse patient outcomes. Sherman et al.demonstrated that hypoxic AECs in APC display increased HIF-1α-dependent signaling, with interleukin-1β(IL-1β)actingas a key inflammatory mediator amplified byHIF-1α activation (11). Pharmacological inhibition of HIF-1α has shown promise in mitigating pulmonary inflammation and limiting APC progression (12). Nucleotide-binding oligomerization domain-like receptor protein 3 (NLRP3) inflammasome, which consists of NLRP3, apoptosis-associated speck-like protein including a CARD (ASC), and pro-Caspase-1, which is involved in the regulation of inflammatory processes by Caspase-1-mediated maturation of IL-18, IL-1β, and IL-33 (13). Recent studies have revealed that HIF-1α in AECs facilitates NLRP3 inflammasome activation following APC, establishing a mechanistic link to ARDS (11). According to these data, targeting the HIF-1α-NLRP3 axis might be a workable way to stop APC from moving into ARDS. However, several limitations still exist in current APC-related research. Although HIF-1α has been demonstrated to participate in APC-associated inflammatory responses (11), the upstream pharmacological regulatory mechanisms targeting this “hypoxia–pyroptosis” axis remain unclear. Most existing studies mainly focus on general anti-inflammatory effects, while the role of HIF-1α-dependent pyroptosis in APC has not been systematically investigated. In addition, the direct regulatory effects of natural compounds on HIF-1α signaling in APC have rarely been reported.

Fraxinellone (FRA), a bioactive phytochemical discovered from the barcinateds of cagazine (Dictamnus dasycarpus) (14), exhibits multiple pharmaceutical activities. FRA has been shown to suppress HIF-1α protein synthesis and Signal Transducer and Activator of Transcription 3 (STAT3) activation, thereby inhibiting tumor angiogenesis and neoplastic growth (15). So far, there have been only a limited number of rigorously designed clinical trials that have concentrated on FRA. Preclinical studies have also demonstrated the FRA-mediated inhibition of neuroblastoma progression through SIRT3 downregulation (16), while other work has documented its anti-fibrotic effects stemming from binding immunoglobulin protein (BIP)-dependent fibroblast inhibition (17). Additional evidence has highlighted FRA’s neuroprotective properties (18–20) and its anti-inflammatory potential, particularly through NLRP3 inflammasome suppression in murine models of colitis (21) and pancreatitis (22). Despite these promising findings, the therapeutic potential of FRA in APC has not yet been examined. Therefore, this study was developed to systematically investigate the protective effects of FRA against APC using complementary *in vivo* and *in vitro* approaches.

## Materials and methods

### Cell culture and reagents

Mouse alveolar epithelial cell line MLE-12 (ATCC^®^ CRL-2110™) was purchased from the American Type Culture Collection (ATCC, Manassas, VA, USA) and kept in Dulbecco’s Modified Eagle Medium (DMEM, Cat#D0819, Seven Biotechnology, Beijing, China) supplemented with 10%heat-inactivated fetal bovine serum (FBS) and 100 U/mL penicillin/streptomycin (Cat#P1400, Solarbio, Beijing, China). Cells were grown in a humidified incubator with 5%CO_2_ at 37 °C. Cells were plated in 6-well dishes at a density of 4 × 10^5^ cells/mL for the experiments. Thrombin (THR, Cat# HY-P0036, MedChemExpress, Shanghai, China) and fraxinellone (FRA, Cat# HY-N0573, MedChemExpress, Shanghai, China) were prepared according to manufacturer instructions.

### Animal models

Male C57BL/6 wild-type (WT) mice, aged 8 to 10 weeks (n = 8 per group), were obtained from the Laboratory Animal Center at Yangzhou University (Production License No. SCXK 2023-0012). The mice were given specific pathogen-free (22 ± 1 °C, 55%humidity, 12 h light/dark cycle) for seven days of acclimatization. APC was induced by weight-drop injury as previously reported (23–26), with modifications. Briefly, mice were anesthetized with isoflurane (5% induction, 1.5% maintenance), and a 50 g stainless steelrod (1.8 cm diameter) was dropped from 30 cm through a vertical guide tube onto the right lateral chest.

Mice were randomly assigned to the following groups: (1) Control group: received an equal volume of vehicle (5% dimethyl sulfoxide (DMSO) in saline) intraperitoneally (i.p.); (2) APC group: received vehicle i.p. 1 day before injury; (3) APC+FRA group: received FRA (1/2.5/5 mg/kg, dissolved in 5% DMSO/saline) i.p. 1 day before injury; (4) FRA group: received FRA (1/2.5/5 mg/kg, dissolved in 5% DMSO/saline) i.p.; (5) APC+FRA+ML228 group: received FRA (5 mg/kg, dissolved in 5% DMSO/saline) and then ML228 (5 mg/kg, dissolved in 5% DMSO/saline) i.p. 1 day before injury.

Lung injury was assessed on chest computed tomography (CT) images using an established semi-quantitative visual scoring method (1). The detailed procedure is described as follows:

Mice were anesthetized and subjected to micro-CT scanning at specific time points (e.g., 6 hours post-injury) by two researchers who were blinded to the experimental group allocation. Each lung lobe was divided into three regions: upper, middle, and lower. Scoring was performed based on the percentage of the area exhibiting abnormal findings (e.g., ground-glass opacity, consolidation) relative to the total area of each region, following the criteria below:

0 point = no abnormalities;
1 point = < 25% abnormal area;
2 points = 25–50% abnormal area;
3 points = 50–75% abnormal area;
4 points = > 75% abnormal area.

The scores of all six regions (three regions per lung for both sides) were summed to generate a total lung injury CT score for each mouse, with a range of 0–24 points. This score was then used for intergroup statistical comparisons. By quantifying the extent of non-aerated lung tissue, this scoring method evaluates the severity of lung injury, which is consistent with the principle underlying the lung contusion volume assessment commonly adopted in clinical practice (2).

All animal experiments received approval from the Institutional Animal Care and Use Committee at Yangzhou University Affiliated Hospital (IACUC No. 202302001) and were conducted in compliance with the NIH Guide for the Care and Use of Laboratory Animals.

### Lung wet/dry weight ratio

At 24 hours post-injury, mice were euthanized by pentobarbital sodium overdose. The whole lung was promptly dissected, and any extraneous tissue or fluid was gently removed with filter paper. The wet weight was measured immediately on an analytical balance. The tissue was then placed in a drying oven at 80 °C for 48 hours until a constant dry weight was achieved. The lung wet-to-dry (W/D) weight ratio was calculated to assess the degree of pulmonary edema.

### Bronchoalveolar lavage fluid (BALF)

Following deep anesthesia, mice were secured in a supine position. The trachea was exposed through a midline cervical incision. A tracheal cannula was inserted through a small incision between the cartilaginous rings and secured in place with a ligature. The lungs were lavaged via the cannula with ice-cold sterile phosphate-buffered saline (PBS), instilling approximately 0.8 mL per wash. After a brief pause of a few seconds, the fluid was slowly withdrawn. This lavage cycle was repeated 3–4 times. The recovered BALF was immediately kept on ice and centrifuged at 800 × g for 10 minutes at 4 °C. The supernatant was aliquoted and stored at –80 °C; the cell pellet was resuspended for further analysis. All steps were performed aseptically with gentle handling on ice to maintain sample integrity.

### Histopathological analysis

Lung tissues were fixed in 4% PFA, embedded in paraffin, and cut into sections with a thickness of 4 μm. Hematoxylin and eosin (HE) staining was used to deparaffinize, dehydrate, and then HE staining for morphological assessment. Immunohistochemistry (IHC) was performed using antibodies against HIF-1α, NLRP3, and gasdermin D (GSDMD) (1:50;Cell Signaling Technology, Shanghai, China) with 3, 3 ′-diaminobenzidine (DAB) as the chromogen. Images were taken with a Nikon Eclipse Ci-L light microscope (Japan). For lung tissue: Fixed, dehydrated, and paraffin-embedded tissues were sectioned (4-5 µm). Following deparaffinization, antigen retrieval was performed, and endogenous peroxidase activity was blocked. Sections were incubated with primary antibody, then HRP-conjugated secondary antibody, developed with DAB, and counterstained with hematoxylin. For MLE-12: Cells cultured on coverslips were fixed, permeabilized, and blocked. Cells were then incubated with primary and fluorescent dye-conjugated secondary antibodies, followed by 4′,6-diamidino-2-phenylindole (DAPI) staining for nuclei. Coverslips were mounted and imaged using a fluorescence microscope.

Histopathological scoring was performed by two independent pathologists blinded to the experimental groups. Lung injury was assessed based on the following criteria: (1) alveolar wall thickening, (2) inflammatory cell infiltration, (3) hemorrhage, and (4) alveolar structure integrity. Each criterion was scored from 0 (normal) to 3 (severe), and the total score (ranging from 0 to 12) was used for statistical comparison between groups.

### Cell viability assay

Based on prior studies demonstrating a link between THR and alveolar epithelial injury, THR was used to induce APC *in vitro* (27–30). Next, in MLE-12 cells, we created a THR-induced injury modelto simulate APC *in vitro*. The successful establishment of the model was confirmed by a comprehensive set of criteria, as commonly adopted in studies of Acute pulmonary contusion cell models (3). Briefly, after exposure to THR (0.8 U/mL for 4 h), compared with the control group: (1) cell viability assessed by CCK-8 assay was significantly reduced; (2) the release of lactate dehydrogenase (LDH) was markedly increased; (3) the levels of pro-inflammatory cytokines (IL-1β, IL-6, TNF-α) in the supernatant were significantly elevated, as measured by ELISA; and (4) the protein expression of key pyroptosis markers (NLRP3, cleaved caspase-1, GSDMD-N) was notably upregulated. These results collectively confirmed the successful induction of an inflammatory cell injury model. In this experiment, drug dosage-response relationships were studied. The Cell Counting Kit-8 (CCK-8, Yeasen, Shanghai, China) was used to measure the vitality of cells. MLE-12 cells were plated in 96-well plates (4 × 10^3^cells/well). The vehicle medium was given in control wells (n = 3 repetitions per group)in equal amounts. Cells were pretreated with FRA (0, 1, 3, 5, 10, 30 μM) or vehiclefor 24 h and subsequently exposed to THR (0.8 U/mL) for 4 h. CCK-8 reagent (10μL/well) was then added, and absorbance was measured at 450 nm (reference 650 nm) using a SpectraMax M5 microplate reader (Molecular Devices, USA).

The THR-induced injury model was established in MLE-12 cells using 0.8 U/mL THR for 4 h, a condition optimized in preliminary experiments and consistent with prior studies of thrombin-mediated epithelial injury (27–30). Subsequently, FRA was tested at three concentrations (2.5, 5, and 10 µM) with a 24 h pretreatment period based on the dose–response relationship established in the CCK-8 assay.

### 5-Ethynyl-2’-deoxyuridine (EdU) uptake assay

For EdU staining, MLE−12 cells were seeded in 6−well plates and pretreated with FRA (2.5, 5, or 10 µM) for 24 h, followed by exposure to THR (0.8 U/mL) for 4 h to induce injury. Cell proliferation was measured using the BeyoClick EdU-488 Kit (10μM; Cat#C0071S, Beyotime, Shanghai, China). Cells were planted into 6-well plates at a density of 4 × 10^5^ cells/well and incubated with 10 μM EdU for 4 h on day 2 post-seeding. After fixation with 4% paraformaldehyde (PFA, pH 7.4) and permeabilization using 0.3%Triton X-100 in PBS, the cells were incubated with the Click reaction cocktail for 30 minutes in the dark. Hoechst 33342 was used to counterstain the nucleus, and pictures were taken with a fluorescence microscope.

### Enzyme-linked immunosorbent assays (ELISAs)

For cytokine measurement, MLE−12 cells were cultured in 6−well plates and subjected to the same treatment protocol: pretreatment with FRA (2.5, 5, or 10 µM) for 24 h, then incubation with THR (0.8 U/mL) for 4 h before supernatant collection. Mouse lung tissue was weighed, homogenized in cold lysis buffer, and centrifuged. The supernatant protein concentration was determined using a BCA assay. Cytokine concentrations were measured using ELISA kits(IL-1β Cat#KE10002, IL-6 Cat#KE10006, IL-18 Cat#KE10012, TNF-α Cat#KE10104; KeyGEN BioTECH) according to the manufacturer’s instructions. Standard curves were established using recombinant cytokines provided in the kits, with a detection range of 15.6–1000 pg/mL. Absorbance was recorded at 450 nm, using a reference wavelength of 570 nm.

### Cell counting in mouse BALF, serum, and blood

For BALF, the collected fluid was centrifuged at 400 × g for 10 min at 4 °C. The cell pellet was resuspended in 1 mL of PBS. For blood, a small volume of whole blood or serum was collected. Total cell counts for all samples were performed using a hemocytometer (e.g., Neubauer chamber) with Trypan Blue stain (0.4%) to distinguish live/dead cells. Alternatively, counts can be obtained using an automated cell counter. Cell concentrations are expressed as cells per milliliter (cells/mL). For differential counts, cytospin preparations of BALF cells are stained (e.g., with Wright-Giemsa) for manual differentiation under a microscope.

MLE-12 were randomly assigned to the following groups: (1) Control group: received an equal volume of vehicle (PBS); (2) THR group: received THR with 0.8 U and 4 hours; (3) THR+FRA group: received FRA FRA (2.5/5/10 μmol/L, dissolved in PBS) 24 hours before injury; (4) FRA group: received FRA (2.5/5/10 μmol/L, dissolved in PBS); (5) negative control(NC)group: received no Hif-1α-siRNA, THR and FRA; (6) Hif-1α-siRNA group: received Hif-1α-siRNA;(7) Control group: received Hif-1α-siRNA, but without THR and FRA.

### Immunofluorescence (IF)

For the detection of specific proteins in MLE-12 cells, immunofluorescence was performed. Cells grown on glass coverslips were fixed with 4% PFA, permeabilized with 0.3% Triton X-100, and blocked with 10% bovine serum albumin (BSA). Subsequently, cells were incubated overnight at 4 °C with the following primary antibodies diluted in blocking buffer: anti-HIF-1α (1:300, Cat# 14179, Cell Signaling Technology), anti-NLRP3 (1:300, Cat# 15101, Cell Signaling Technology), and anti-GSDMD (1:300, Cat# 39754, Cell Signaling Technology). After washing, Alexa Fluor 488- or 594-conjugated secondary antibodies (1:300, Beyotime, Shanghai, China) were applied for 1 hour at room temperature. Nuclei were counterstained with DAPI. Fluorescence images were captured using a Zeiss LSM 900 confocal microscope (Germany).

Cell death in lung tissue sections was assessed using a terminal deoxynucleotidyl transferase dUTP nick end labeling (TUNEL) assay kit (Cat# C1086, Beyotime, Shanghai, China) according to the manufacturer’s protocol. Briefly, deparaffinized sections were incubated with Proteinase K (20 μg/mL), labeled with FITC-dUTP, and counterstained with DAPI. The number of TUNEL-positive cells was quantified in five randomly chosen microscopic fields per sample using ImageJ software (National Institutes of Health, USA).

### Quantitative real-time polymerase chain reaction (RT-qPCR)

Total RNA was extracted from MLE-12 cells or mouse lung tissues using the RT6 cDNA Synthesis Kit (TSK302M, Beijing, China) and TRIzol (Beyotime) to reverse transcribe it into cDNA.Using the SYBR Green Master Mix(TSE202, TSINGKE, USA), RT-qPCR was carried out on a ProFlex PCR System(Applied Biosystems, USA). The following primer sequences were used:

- Glyceraldehyde-3-phosphate dehydrogenase (GAPDH): F 5′-ACAGTCAGCCGCATCTTC-3′, R 5′-CTCCGACCTTCACCTTCC-3′
- HIF-1α: F 5′-TGGACTTGTCTCTTTCTCCGC-3′, R 5′-CGACGTTCAGAACTCATCCT-3′
- NLRP3: F 5′-ATTACCCGCCCGAGAAAGG-3′, R 5′-TCGCAGCAAAGATCCACACAG-3′
- GSDMD: F 5′-GATGAGATGTCTCGGCTGCTTG-3′, R 5′-AGCCGTTACGGATATGGTGGTC-3′

### Western blotting

Protein lysates were extracted from MLE-12 cells or mouse lung tissues using RIPA lysis buffer containing protease and phosphatase inhibitors. Protein concentrations were determined using a BCA assay kit. Equal amounts of protein (typically 20-30 µg per lane) were separated by 12% Sodium dodecyl sulfate–polyacrylamide gel electrophoresis (SDS-PAGE) and subsequently transferred onto PVDF membranes. After blocking with 5% non-fat milk for 1 hour at room temperature, the membranes were incubated overnight at 4 °C with the following primary antibodies: anti-HIF-1α (1:1000, Cat# 14179, Cell Signaling Technology), anti-GSDMD (1:300, Cat# 39754, Cell Signaling Technology), anti-NLRP3 (1:1000, Cat# 15101, Cell Signaling Technology), and anti-GAPDH (1:5000, Cat# 5174, Cell Signaling Technology). Following washes, the membranes were incubated with an HRP-conjugated anti-rabbit IgG secondary antibody (1:4000, Cat# 7074, Cell Signaling Technology) for 1 hour at room temperature. Protein bands were visualized using Clarity™ ECL Substrate (Cat# 1705060, Bio-Rad, USA) and quantified using Image Lab 6.1 software (Bio-Rad).

### Biotin-streptavidin pull-down assay

Biotin-labeled fraxinellone (Biotin-FRA) was synthesized by conjugating biotin to FRA via a PEG4 linker (MedChemExpress, Shanghai, China). MLE-12 cells were treated with thrombin (0.8 U/mL) for 4 h to induce HIF-1α expression. Cells were then lysed in RIPA buffer containing protease inhibitors, and the lysates were centrifuged at 12,000 × g for 15 min at 4 °C. Equal amounts of protein (500 μg) were incubated with Biotin-FRA (10 μM)-conjugated streptavidin magnetic beads (Thermo Fisher Scientific, USA) at 4 °C overnight with gentle rotation. For the competitive inhibition group, unlabeled free FRA (100 μM) was added 30 min prior to Biotin-FRA incubation. The beads were washed five times with ice-cold TBS containing 0.1% Triton X-100, and bound proteins were eluted by boiling in SDS-PAGE loading buffer for 5 min. The eluted proteins were subjected to Western blot analysis using anti-HIF-1α antibody (1:1000, Cat# 14179, Cell Signaling Technology). Input samples (30 μg total protein) were run in parallel as loading controls.

### HIF-1α siRNA knockdown experiment

MLE-12 cells were transfected with HIF-1α-specific siRNA (or a non-targeting scrambled siRNA as a negative control) using Lipofectamine 3000 according to the manufacturer’s protocol. After 6 hours, the transfection medium was replaced with fresh complete medium. Cells were then cultured for an additional 24–48 hours to allow for target gene knockdown before being subjected to subsequent treatments or analyses. Knockdown efficiency was confirmed by Western blotting.

### Molecular docking

The 3D structure of FRA (CID 3083584) was retrieved from PubChem and underwent energy minimization in Chem3D 20.0 using the MM2 force field. The HIF-1α protein structure (UniProt ID: Q61221) was retrieved from the AlphaFold Database. Docking simulations were conducted using AutoDock Vina 1.1.2, with a grid box of 20×20×20 Å centered on the protein’s active site. Hydrogen bonding interactions were seen utilizing PyMOL 2.5 (Schranging, USA).

### Generation of HIF-1α R258S point mutation

The HIF-1α R258S point mutation was introduced into the expression plasmid containing the wild-type Hif1agene using a site-directed mutagenesis kit, with mutation-specific primers designed according to the manufacturer’s instructions. The plasmid sequence was verified by Sanger sequencing. MLE-12 cells were then transfected with either the wild-type or mutant HIF-1α plasmid using Lipofectamine 3000. Stable cell lines were selected with puromycin, and single clones were expanded. Successful expression of the mutant protein and the substitution of arginine (Arg) with serine (Ser) at position 258 were confirmed by Western blot analysis.

### Scanning electron microscopy (SEM)

MLE-12 cells or mouse lung tissues were fixed in 2.5% glutaraldehyde (4 °C, 24 h), post-fixed in 1% osmium tetroxide for 2 h at room temperature, and dehydrated through a graded ethanol series (30%, 50%, 70%, 90%, 100%). Samples were polymerized at 60 °C for 48 hours after being embedded in EPON 812 epoxy resin. Using a Leica UC7 ultramicrotome (Germany) and double-stained with 2% uranyl acetate and Reynolds lead citrate, ultrathin sections (70 nm) were created. Imaging was conducted with a ZEISS Sigma 300 field-emission scanning electron microscope(Oberkochen, Germany) set to 5 kV.

### Statistical analysis

Using the Shapiro-Wilk test, normality was assessed, and the Levene’s test was used to determine variance homogeneity. Using one-way Analysis of Variances (ANOVAs), multiple group comparisons were carried out, followed by the Tukey’s *post hoc* test. A two-tailed p < 0.05 was considered statistically significant.

## Results

### FRA alleviates weight-drop-induced APC in mice

**Figure 1:**
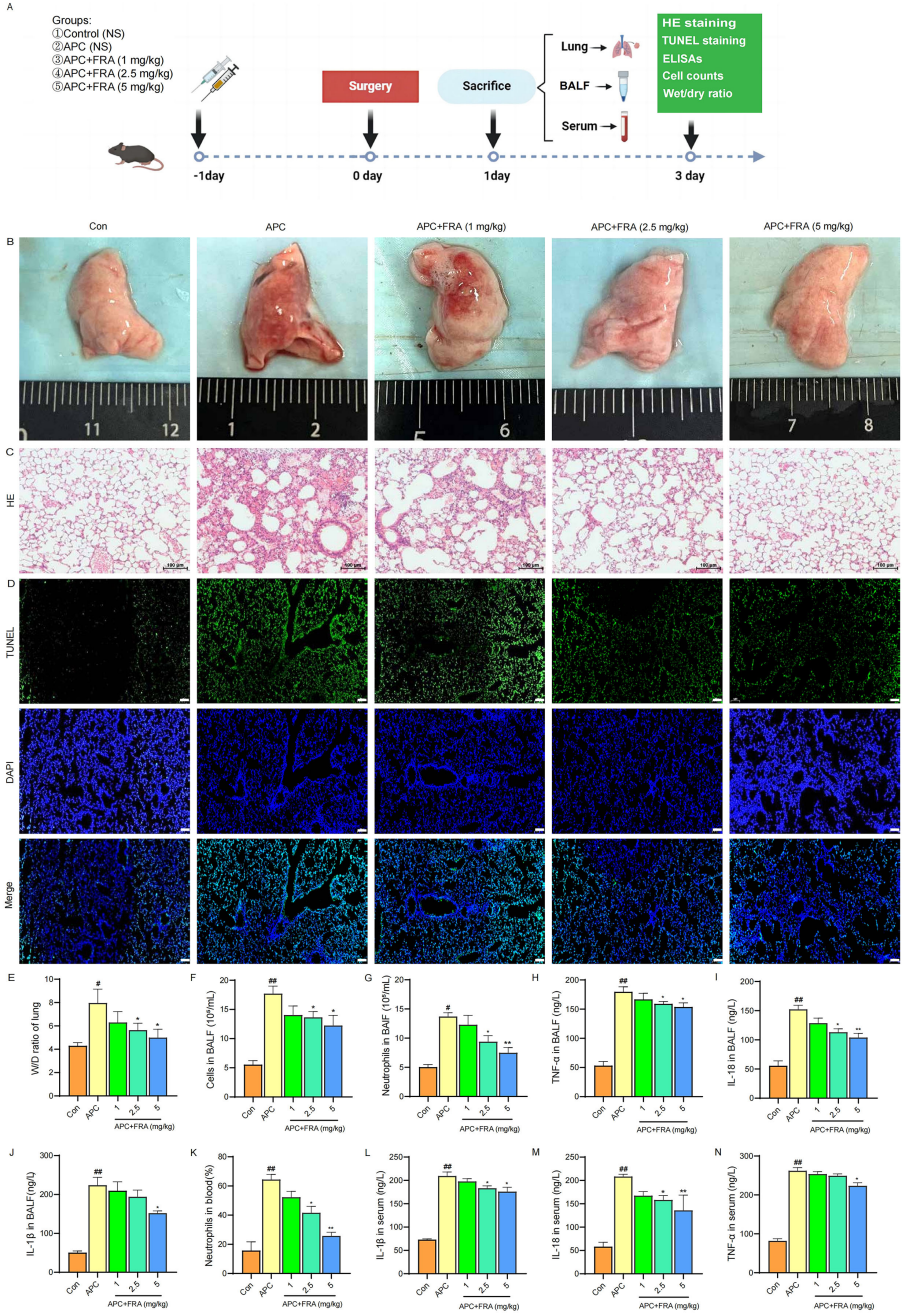
FRA reduces weight-drop-induced APC in mice. **(A)** Flowchart of the mouse treatment protocol. **(B)** Representative images of lung tissues. Lung samples (n = 6/group) were collected for histological evaluation following weight-drop modeling. **(C)** Representative HE staining of lung tissues. **(D)** Representative TUNEL fluorescence staining of lung tissues. Scale bar: 50 μm. **(E)** Lung W/D weight ratio. **(F–G)** Total cell and neutrophil counts in BALF. **(H–J)** IL-1β, IL-18, and TNF-α concentrations in BALF. **(K)** Neutrophil counts in blood. **(L–N)** IL-1β, IL-18, and TNF-α concentrations in serum. Data are presented as mean ± SD (n = 6/group). ^*/#^p < 0.05, ^**/##^p < 0.01. #versus control group, * versus APC group.

The protective effects of FRA were initially evaluated in the weight-drop-induced APC mouse model (**Figure 1A**). Compared with control mice, APC model mice exhibited pronounced pulmonary edema and hyperemia (**Figures 1B, C**). FRA treatment (2.5 mg/kg or 5 mg/kg) dramatically decreased weight-drop-induced cell death (**Figure 1D**), decreased the lung W/D ratio (**Figure 1E**), reduced the number of damaged cells (**Figures 1F, G**), and reduced the levels of IL-1β, IL-18, and TNF-α in BALF (**Figures 1H–J**). The amounts of serum cytokines (**Figures 1K–N**) showed similar decreases. Together, these findings indicate that FRA substantially reduces inflammation and pathological alterations in the lung tissues of APC mice. In subsequent experiments, mice in the treatment group received intraperitoneal injections of FRA (5 mg/kg) (17, 18) 1 day before injury, followed by induction of APC.

### FRA suppresses the expression of protein expression caused by weight drop in mice with pyroptosis

**Figure 2:**
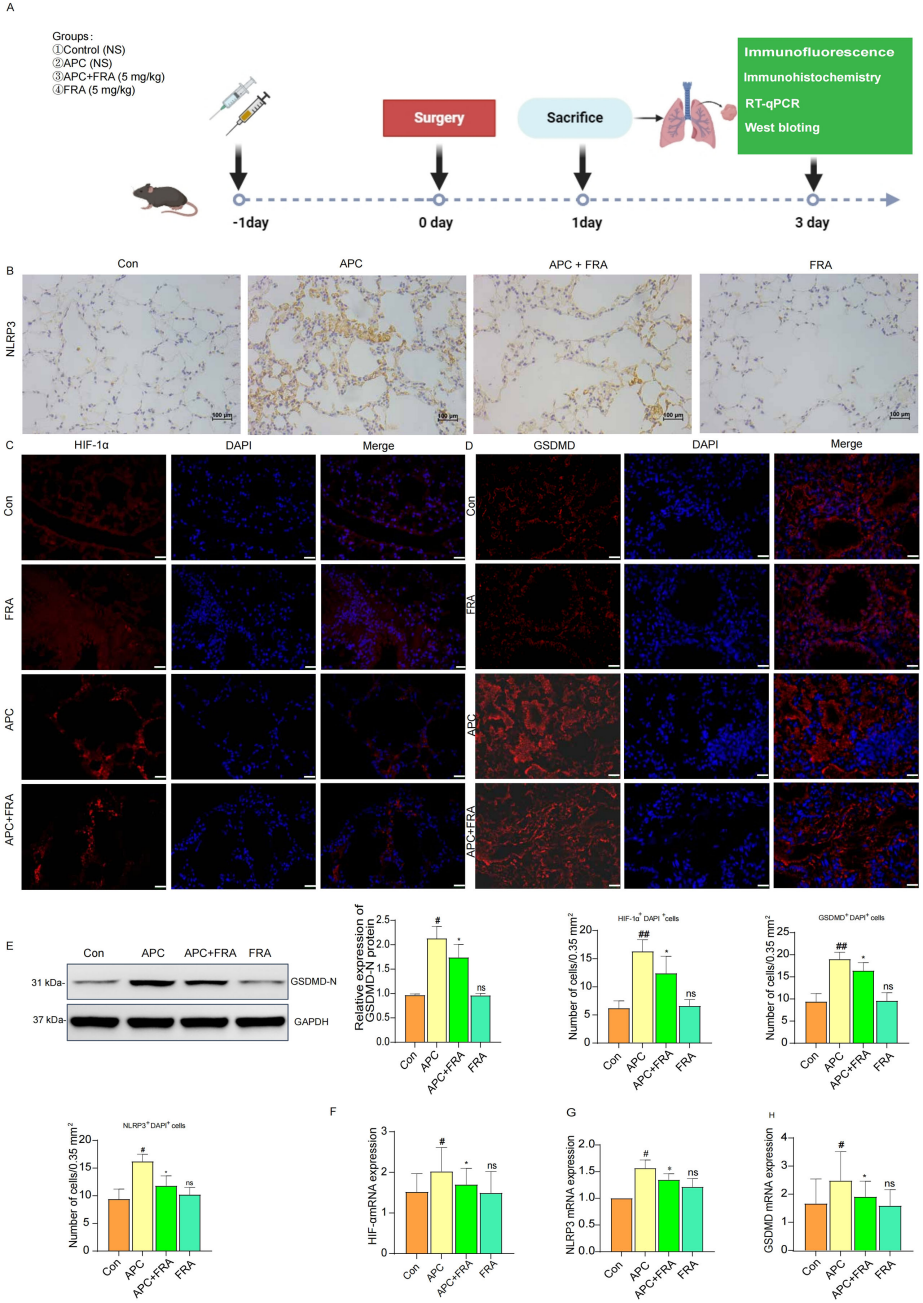
FRA inhibits the weight-drop-induced expression of pyroptosis-related proteins in mice. **(A)** Flowchart of the mouse treatment protocol. **(B)** Representative IHC staining results for NLRP3. **(C, D)** Representative immunofluorescence images of HIF-1α and GSDMD. Scale bar: 50 μm. **(E)** Western blotting analysis of GSDMD-N. **(F–H)** Expression levels of HIF-1α, NLRP3, and GSDMD as determined by RT-qPCR (GAPDH as internal reference). Data are presented as mean ± SD (n = 6/group). ns p>0.05, #/*p<0.05, ##p<0.01. #/ns versus control group, * versus APC group.

In the weight-drop-induced APC model, pyroptosis was assessed by evaluating the expression of HIF-1α, NLRP3, and GSDMD. As shown in **Figure 2**, the levels of these three proteins were markedly elevated in APC mice compared with controls. Significant inhibition was seen by FRA therapy to the expression of these proteins related to pyroptosis (**Figures 2A–D**). Western blotting was used to get results that were consistent (**Figure 2E**) and RT-qPCR analyses (**Figures 2F–H**).

### FRA attenuates weight drop-induced APC-associated pyroptosis in mice by downregulating HIF-1α

**Figure 3:**
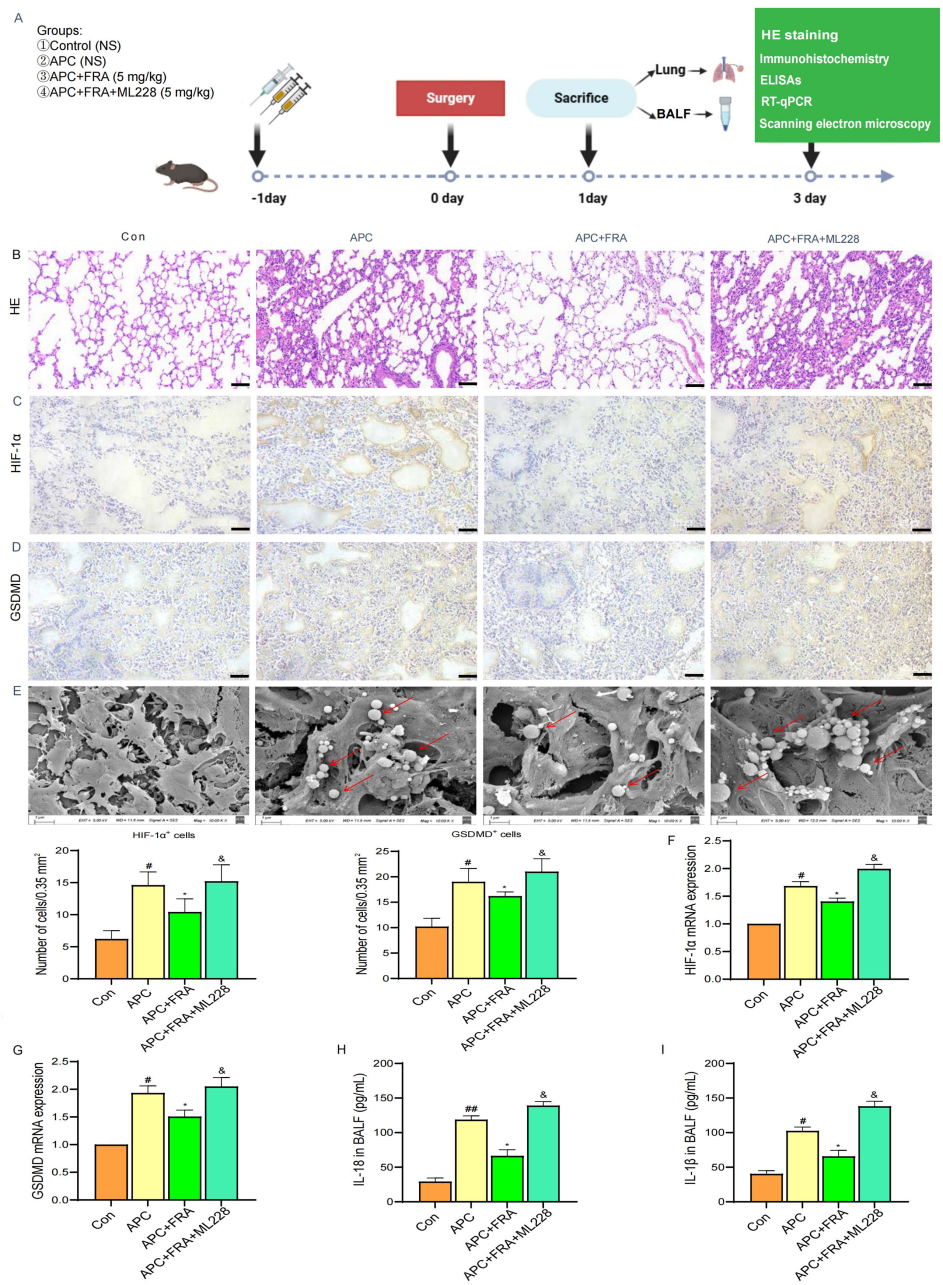
FRA attenuates pyroptosis associated with weight-drop-induced APC in mice through HIF-1α downregulation. **(A)** Flowchart of the mouse treatment protocol. **(B)** Representative HE staining of lung tissues. **(C, D)** Representative IHC of HIF-1α and GSDMD. Scale bar: 50 μm. **(E)** SEM of lung tissues. Scale bar: 1 μm. Red arrows indicate pyroptotic bodies in lung tissues. **(F, G)** Expression levels of HIF-1α and GSDMD determined by RT-qPCR (GAPDH as internal reference). **(H, I)** IL-1β and IL-18 concentrations in BALF. Data are presented as mean ± SD (n = 6/group). #/*/&p<0.05, ##p<0.01. #versus control group, * versus APC group, & versus APC+FRA group.

**Figure 4:**
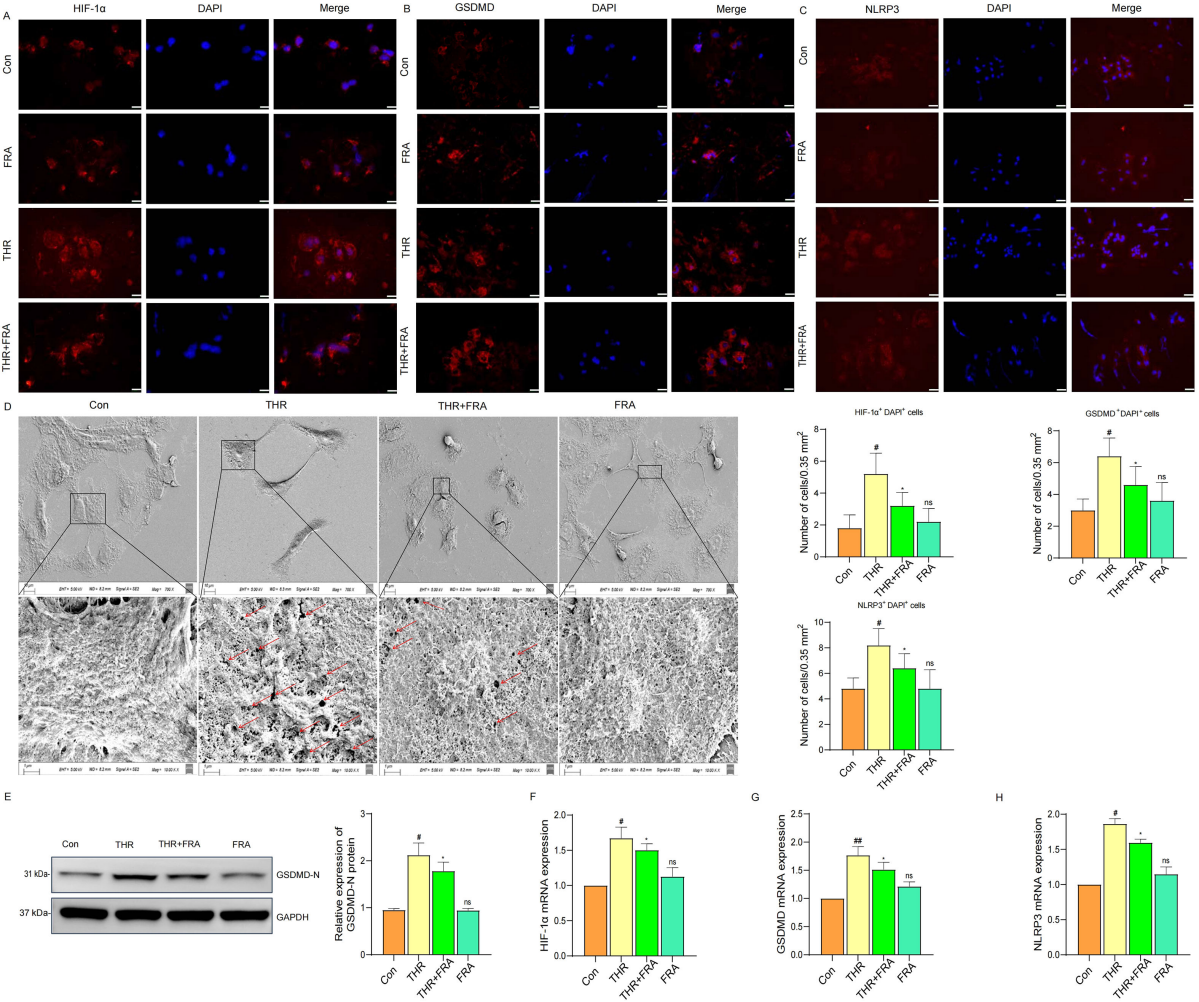
FRA inhibits the expression of pyroptosis-related proteins induced by THR in MLE-12 cells. **(A–C)** Representative immunofluorescence images of HIF-1α, NLRP3, and GSDMD. Scale bar: 50 μm. **(D)** SEM of MLE-12 cells. Scale bar: 50 μm (upper panel) and 1 μm (lower panel). Red arrows indicate membrane perforations caused by pyroptosis. **(E)** Western blotting analysis of GSDMD-N. **(F–H)** Expression levels of HIF-1α, NLRP3, and GSDMD determined by RT-qPCR (GAPDH as internal reference). Similar results were obtained from three independent experiments. All data are presented as mean ± SD ns p>0.05, #/*p<0.05, ##/**p<0.01. #/ns versus control group, * versus THR group.

To explore whether FRA’s protective effects against APC are mediated by the downregulation of HIF-1α, mice were pretreated with the HIF-1α activator ML228 (5 mg/kg) for one day, as indicated by a prior study (28), before receiving FRA administration (**Figure 3A**). As shown in **Figure 4**, ML228 significantly reversed the protective effects of FRA on APC (**Figure 3B**). IHC staining confirmed that ML228 upregulated HIF-1α and GSDMD at the protein level in the lungs, counteracting the effects of FRA (**Figures 3C, D**). SEM analysis further demonstrated that the levels of pyroptotic bodies in lung tissues were markedly increased in both the APC and APC+FRA+ML228 groups, whereas FRA alone diminished this effect (**Figure 3E**). Similar results were observed for HIF-1α, NLRP3, and GSDMD mRNA expression (**Figures 3F–G**) and for inflammatory cytokine responses (**Figures 3H–I**).

### MLE-12 cells THR-induced APC is decreased by FRA

To examine the protective role of FRA (**Supplementary Figure 1A**), cells were pre-incubated with different concentrations of FRA for 12, 24, and 48 h prior to THR exposure (29). **Supplementary Figure 1B** and **Supplementary Figure 2** shows the changes in MLE-12 cell viability induced by FRA treatment in a time- and concentration-dependent manner. FRA treatment significantly improved cell proliferation, with 10 μM FRA for 24 h showing the most robust effect (**Supplementary Figure 1C**). FRA also decreased the amount of IL-6, TNF-a, and IL-18 secretion in a way that was dose-dependent (**Supplementary Figures 1D–F**). EdU staining confirmed that FRA enhanced cell proliferation following THR exposure (**Supplementary Figure 1G**). A 10 μM FRA concentration with a 24 h treatment duration and 0.8 u/ml THR with a 4 h treatment length was chosen for the following experiments because of these results. Overall, these data show that FRA guards against MLE-12 cells from APC caused by THR.

### In MLE-12 cells, FRA prevents the expression of protein related to THR-induced pyroptosis.

In the THR-induced APC model, pyroptosis was assessed by measuring HIF-1α, NLRP3, and GSDMD expression. As shown in **Figure 4**, these proteins were markedly upregulated in the THR group compared with controls. The treatment with FRA successfully reduced their expression (**Figures 4A–C**). SEM analysis demonstrated that THR markedly increased membrane perforations in MLE-12 cells, while FRA alleviated this phenotype (**Figure 4D**). Consistent results were achieved through Western blotting (**Figure 4E**) and RT-qPCR (**Figures 4F–H**).

### FRA alleviates THR-induced pyroptosis in MLE-12 cells through specific binding to HIF-1α

**Figure 5:**
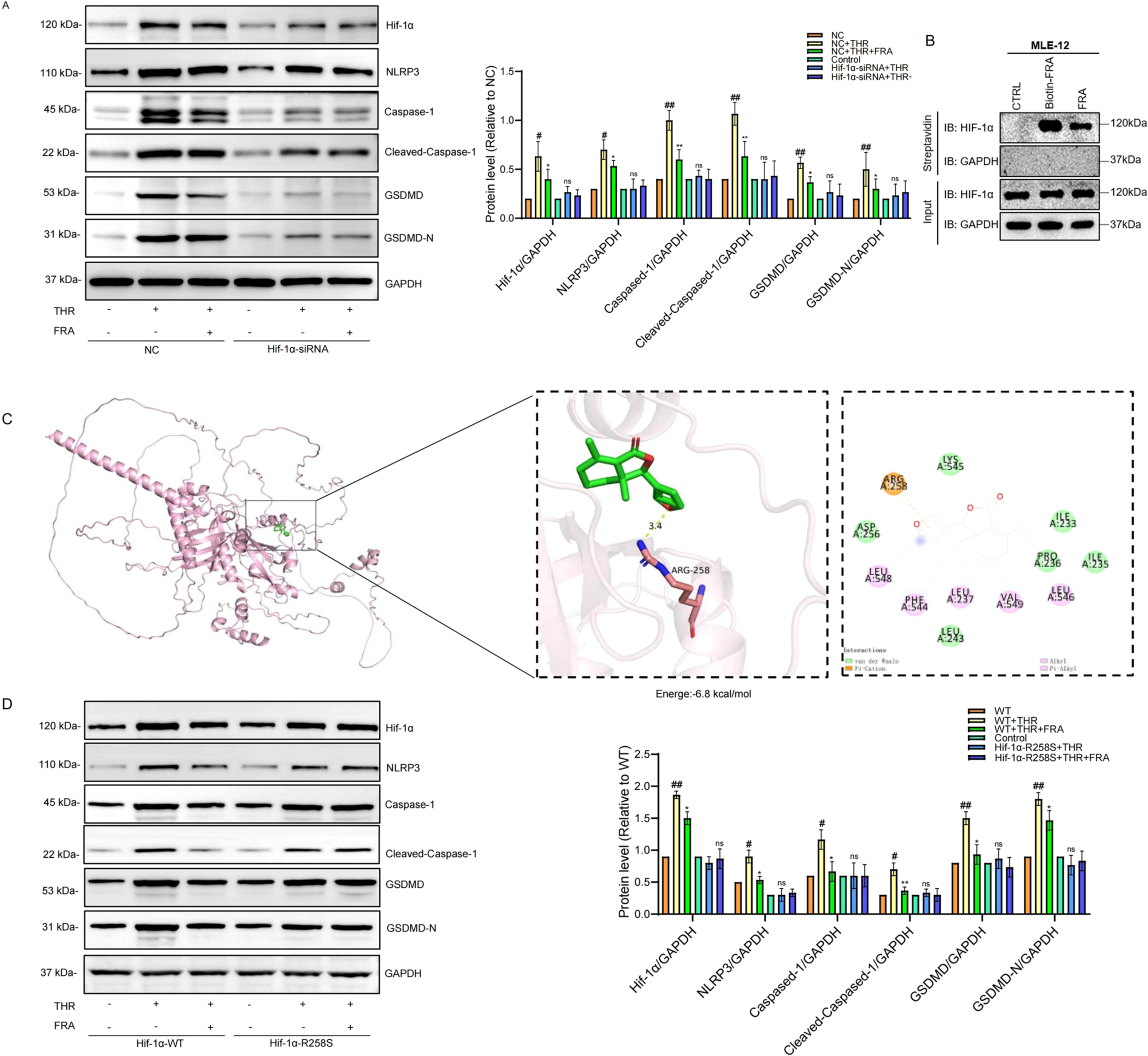
FRA alleviates THR-induced pyroptosis in MLE-12 cells through specific binding to HIF-1α. **(A)** Western blot analysis of pyroptosis-related proteins following HIF-1α silencing under various treatments. **(B)** The pull down assay between FRA and HIF-1α by using biotin-labeled FRA (Biotin-FRA). **(C)** Molecular docking of FRA with HIF-1α. **(D)** Western blotting analysis of pyroptosis-related proteins following HIF-1α mutation under various treatments. Data are presented as mean ± SD (n = 6/group). #/*p<0.05, ##/**p<0.01, ns p>0.05. # versus negative control (NC) group, *versus NC-THR group, ns versus control group.

**Figure 6:**
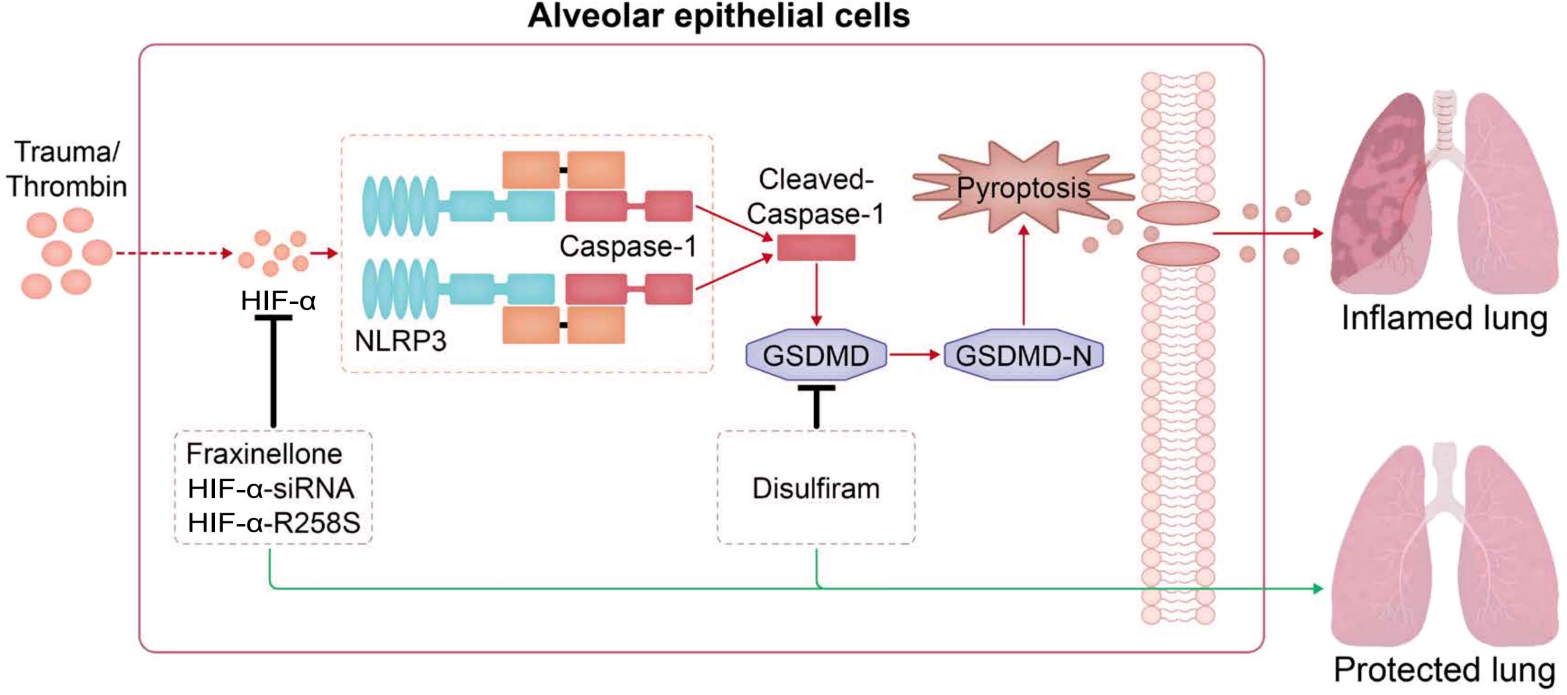
Schematic diagram illustrating the mechanism by which FRA alleviates APC.

Expressed with the negative control (NC) group, no significant differences were seen in the expression of pyroptosis-associated proteins in the HIF-1α-siRNA group, regardless of FRA treatment(**Figure 5A**). To further validate this predicted interaction biochemically, we performed a biotin-streptavidin pull-down assay using Biotin-FRA and MLE-12 cell lysates. As shown in **Figure 5B**, Biotin-FRA successfully pulled down endogenous HIF-1α protein, whereas the control group (without Biotin-FRA) showed no detectable HIF-1α signal. Notably, pre-incubation with an excess of unlabeled free FRA competitively abolished this interaction, confirming the specificity of FRA binding to HIF-1α. These results provide direct biochemical evidence that FRA physically interacts with endogenous HIF-1α protein in MLE-12 cells. Molecular docking analysis indicated that FRA binds directly to HIF-1α at the Arg258 residue (**Figure 5C**). To validate this, MLE-12 cells were transfected with either wild-type (WT) HIF-1α or a mutant vector (R258S, Arg→Ser) (**Figure 5D**). There were no notable changes in the mutant group with regard to protein expression associated with pyroptosis, regardless of FRA treatment. Taken together, these findings demonstrate that FRA inhibits NLRP3/GSDMD inflammasome activation by binding specifically to the Arg258 residue of HIF-1α. A schematic illustration of FRA-mediated alleviation of APC is shown in **Figure 6**.

## Discussion

APC is characterized by severe hypoxemia and pulmonary manifestations of multiple organ dysfunction syndrome, often progressing to uncontrolled, self-perpetuating lung inflammation (31, 32). The high prevalence of this condition and associated mortality among critically ill patients continues to impose a substantial public health burden (33, 34). Despite advances in the understanding of APC pathogenesis, therapeutic options for affected patients remain limited, highlighting the urgent need for novel strategies.This study revealed that FRA inhibits HIF-1α activity and reduces pyroptosis in experimental APC models, making it a promising candidate for treating this severe condition.

FRA has received attention for its anti-inflammatory and antioxidant properties (15 21), yet its potential function in APC treatment is poorly defined. Pyroptosis, a form of programmed cell death, has been linked to the progression of ALI (35–37). In this study, we established both *in vivo* (weight-drop-induced lung injury in mice) and *in vitro* (THR-induced injury in MLE-12 cells) APC models, based on established methodologies (27–30). We observed that weight-drop injury and THR exposure induced pyroptosis in lung tissues and epithelial cells, whereas pyroptosis inhibitors attenuated APC in this experimental context. Notably, ischemia-reperfusion injury (IRI) also involves pyroptosis as a primary cell death pathway, and NLRP3 inhibitors have been shown to reduce infarct size via the suppression of NLRP3 activity (38–40). In our models, FRA reduced lung W/D ratio, and pro-inflammatory cytokine release, all correlating with APC severity. In addition, FRA decreased the accumulation of HIF-1α and the activation of NLRP3 inflammasomes. Importantly, FRA attenuated weight-drop-induced pulmonary edema, protein cast formation, and pathological changes, without observable toxicity at therapeutic doses. To our knowledge, this study provides new evidence that FRA mitigates APC, at least in part, through modulation of pyroptosis-associated pathways.

We further characterized the molecular mechanisms underlying the effects through which FRA protects against APC. Current evidence indicates that hypoxia-sensitive type II AECs are major drivers of inflammation via HIF-1α activation (41). APC pathogenesis involves systemic hypoxia-induced HIF-1α signaling, leading to elevated pro-inflammatory cytokine release, including IL-1β and IL-6 (41). Consistent with this, our data showed that HIF-1α activation with ML228 exacerbated lung edema, pyroptosis, and cytokine release in APC models, while HIF-1α knockdown significantly reduced pyroptotic responses, in line with findings published by Suresh et al. (42). Furthermore, molecular docking analysis revealed that FRA binds specifically to the HIF-1α Arg258 residue, linking its inhibitory effects on THR-induced pyroptosis to suppression of the NLRP3/GSDMD pathway. All of them point to the treatment possibility of focusing on HIF-1α in order to slow the development of APC.

Therapeutic efficacy is the top priority in order to choose the best clinical medication, as this will reduce the negative effects of the drug. Although pharmacological benefits often increase in a dose-dependent manner, parallel escalation of side effects frequently limits clinical applicability. Importantly, our investigation demonstrated that FRA exhibited an exceptional safety profile at therapeutically effective concentrations across multiple experimental models. The current approach to managing APC is primarily nonspecific, relying on mechanical ventilation, volume resuscitation, glucocorticoids, and pulmonary vasodilators, often in combination with anti-inflammatory agents like aspirin, salbutamol, and ketoconazole (43, 44). Nonetheless, there is insufficient data to support the improvement of patient outcomes by these therapies. Increasing evidence implicates hypoxia signaling through HIF-1α as a central driver of APC pathogenesis. The FRA was shown to be a unique HIF-1α inhibitor by our research, which also successfully reduces the pyroptosis and inflammatory cascades caused by weight and THR in APC models. As a result, FRA is suggested as a possible option for translational therapeutic development.

Despite previous reports demonstrating the anti-inflammatory and NLRP3-inhibitory effects of FRA (22), its role appears to be highly context-dependent across different disease models, and its function in trauma-associated APC has not been systematically investigated. The present study extends existing knowledge by addressing this specific gap. First, our study provides a comprehensive evaluation of FRA in an APC model, which is mechanistically distinct from previously studied inflammatory conditions such as colitis, pancreatitis, or cancer. APC is characterized by mechanical injury combined with hypoxia-driven secondary inflammation, representing a more complex pathophysiological context (11). Our findings demonstrate that FRA confers significant protective effects in this trauma-induced lung injury model, thereby expanding its potential therapeutic scope. Second, we identify and functionally validate a hypoxia-responsive pyroptosis regulatory axis involving HIF-1α and NLRP3/GSDMD signaling. While prior studies have shown that FRA can modulate inflammatory pathways (20–22), whether it regulates pyroptosis through HIF-1α-dependent mechanisms has remained unclear. In this study, we establish a causal relationship by integrating pharmacological activation (ML228) and genetic silencing (siRNA), demonstrating that FRA-mediated protection is closely associated with suppression of HIF-1α-driven pyroptotic signaling. Third, our data suggest a potential target-specific mechanism whereby FRA interacts with HIF-1α at the Arg258 residue. Although molecular docking and site-directed mutagenesis do not fully substitute for direct biochemical binding assays, these findings provide preliminary evidence supporting a more specific mode of action beyond general anti-inflammatory effects. This observation offers a new perspective for understanding how FRA modulates hypoxia-related signaling pathways. Taken together, our study not only broadens the application of FRA in lung injury but also highlights the interplay between hypoxia signaling and pyroptosis as a mechanistic framework. These findings provide a rationale for targeting the HIF-1α–NLRP3/GSDMD axis in trauma-associated pulmonary injury.

Even with these new discoveries about HIF-1α-targeted therapy for APC, there are a lot of drawbacks that need to be made clear. First, our experimental models primarily examined acute-phase pathological manifestations (24–72 h post-induction), which may overlook chronic adaptation mechanisms and long-term therapeutic efficacy. Moreover, translational relevance to clinically achievable dosing regimens remains unverified due to temporal constraints, warranting dose-escalation studies compliant with biomedical ethics. Second, although ML228-mediated HIF-1α activation in mice and siRNA-mediated knockdown in MLE-12 cells produced methodologically valid methods, they may not completely capture the complexity of hypoxia signaling dynamics in human alveolar epithelium.

In addition, several limitations regarding the mechanistic validation of FRA-HIF-1α interaction should be acknowledged. In this study, molecular docking was performed to predict the binding mode, key interaction residues and binding affinity between FRA and HIF-1α, providing credible in silico structural evidence for their stable molecular interaction. Nevertheless, due to objective experimental constraints and limited revision time, we did not perform biochemical validation using Co-IP or pull-down assays based on MLE-12 cells or mouse lung tissues. Hence, direct biological evidence verifying their endogenous physical binding is still lacking. Additionally, the potential binding sites were merely predicted through computational simulation without site-directed mutagenesis verification. Therefore, further biochemical experiments including Co-IP combined with amino acid site mutation will be conducted in our future research to confirm their physical interaction and clarify the precise binding mechanism.

Third, our investigation focused on the NLRP3/GSDMD axis, whereas other pyroptosis-related pathways (e.g., AIM2/Caspase-8) and potential crosstalk with apoptosis or ferroptosis pathways remain unexplored. Moreover, the translational relevance is constrained by limited pharmacokinetic characterization, and critical parameters such as tissue distribution, metabolic stability, and potential off-target effects in primates remain to be systematically evaluated. Finally, the present validation was restricted to weight-drop- and THR-induced APC models, whereas clinical APC frequently coexists with comorbidities such as sepsis or ARDS, emphasizing the need for future studies employing polymorbidity animal models. Moreover, this study specifically evaluated pre-APC-incident medication timing. The therapeutic potential of FRA at other temporal phases remains unexamined and warrants chronotherapy-focused investigation. Notwithstanding these limitations, our study establishes a foundational framework for subsequent investigations into HIF-1α modulation in hypoxia-associated pulmonary pathologies.

## Conclusion

In summary, FRA confers robust protection against weight-drop- and THR-induced APC by attenuating histopathological injury, inflammatory cell infiltration, and pulmonary edema. Mechanistically, FRA binds to HIF-1α at Arg258, thereby suppressing NLRP3/GSDMD-mediated pyroptosis and downstream inflammatory responses. All of them point to FRA as a potentially useful focused treatment candidate for APC, this is justified by the fact that more research is needed in clinical settings.

## Data Availability

The original contributions presented in the study are included in the article/**Supplementary Material**. Further inquiries can be directed to the corresponding authors.

## Supplementary material

Supplementary Figure 1 FRA reduces THR-induced APC in MLE-12 cells. **(A)** Chemical structure of FRA. **(B, C)** Viability of THR-induced MLE-12 cells treated with or without FRA, assessed by CCK-8 assay. **(D–F)** ELISA detection of TNF-α, IL-18, and IL-6 in MLE-12 cells. **(G)** Newly synthesized DNA in MLE-12 cells detected by EdU staining. Data are presented as mean ± SD, ^*/#^p < 0.05, ^**/##^p < 0.01. # versus control group, * versus THR group.

Supplementary Figure 2 Dose-response curves of FRA treatment at different exposure times. Cells were treated with increasing concentrations of FRA (0–30 μmol/L) for 12, 24, and 48 h, and cell viability was assessed using the CCK-8 assay. FRA exhibited both concentration-dependent and time-dependent inhibitory effects on cell viability. The dashed line indicates 50% cell activity, which was used to estimate the approximate half-maximal inhibitory concentration (IC50) values for each treatment duration. Based on these results, 2.5, 5, and 10 μM were selected for subsequent mechanistic experiments to avoid excessive cytotoxicity.
