# Supplementary figures and images for: Fraxinellone mitigates acute lung injury by targeting HIF-1α to suppress pyroptosis and inflammation
Source: Front Immunol. 2026 Jul 16;17:1826165. doi: 10.3389/fimmu.2026.1826165 (PMC13421439; doi:10.3389/fimmu.2026.1826165)

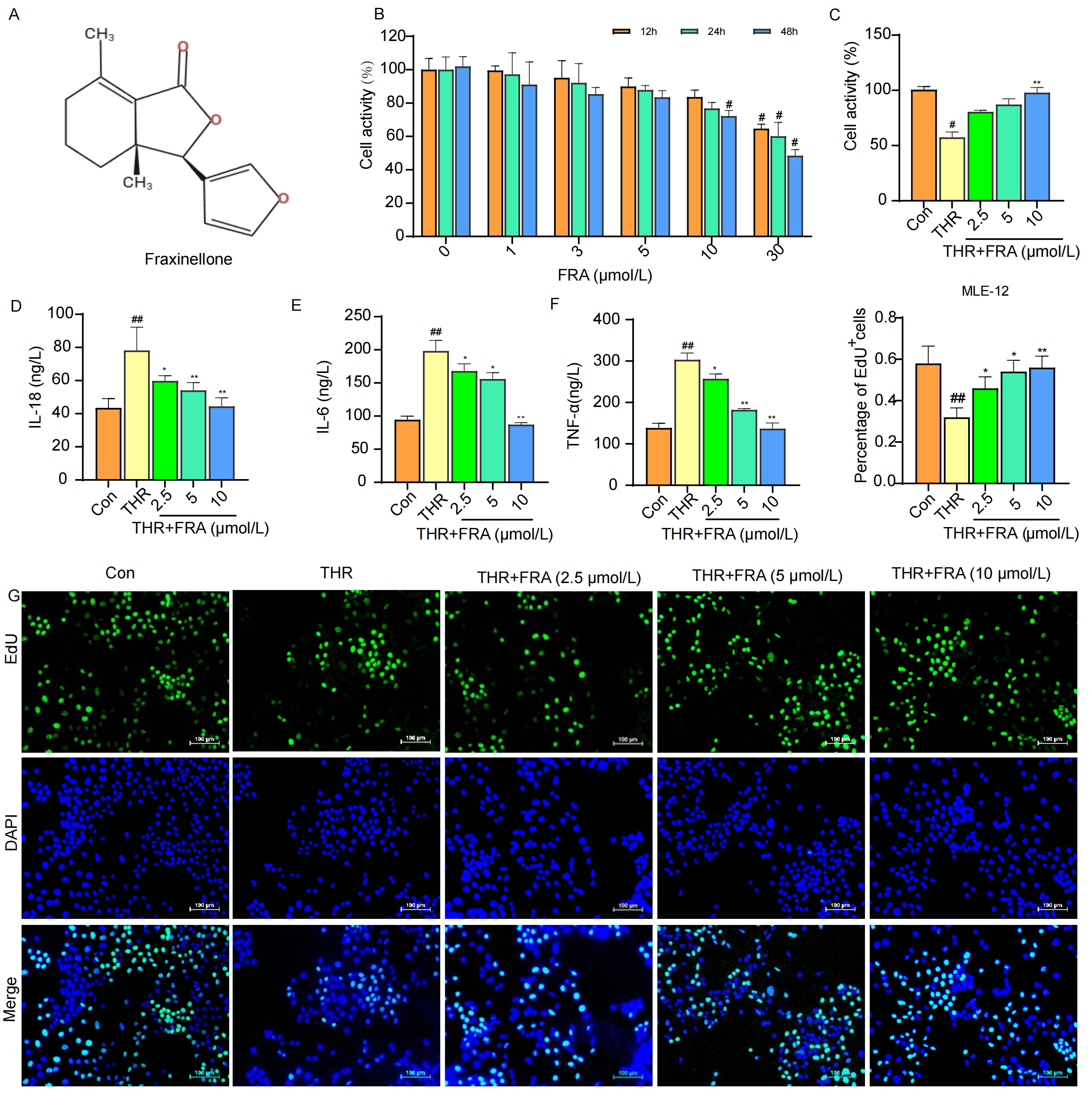

Supplement: Supplementary Figure 1 — FRA reduces THR-induced APC in MLE-12 cells. (A) Chemical structure of FRA. (B, C) Viability of THR-induced MLE-12 cells treated with or without FRA, assessed by CCK-8 assay. (D–F) ELISA detection of TNF-α, IL-18, and IL-6 in MLE-12 cells. (G) Newly synthesized DNA in MLE-12 cells detected by EdU staining. Data are presented as mean ± SD, */#p < 0.05, **/##p < 0.01. # versus control group, * versus THR group. [file Image1.jpeg]

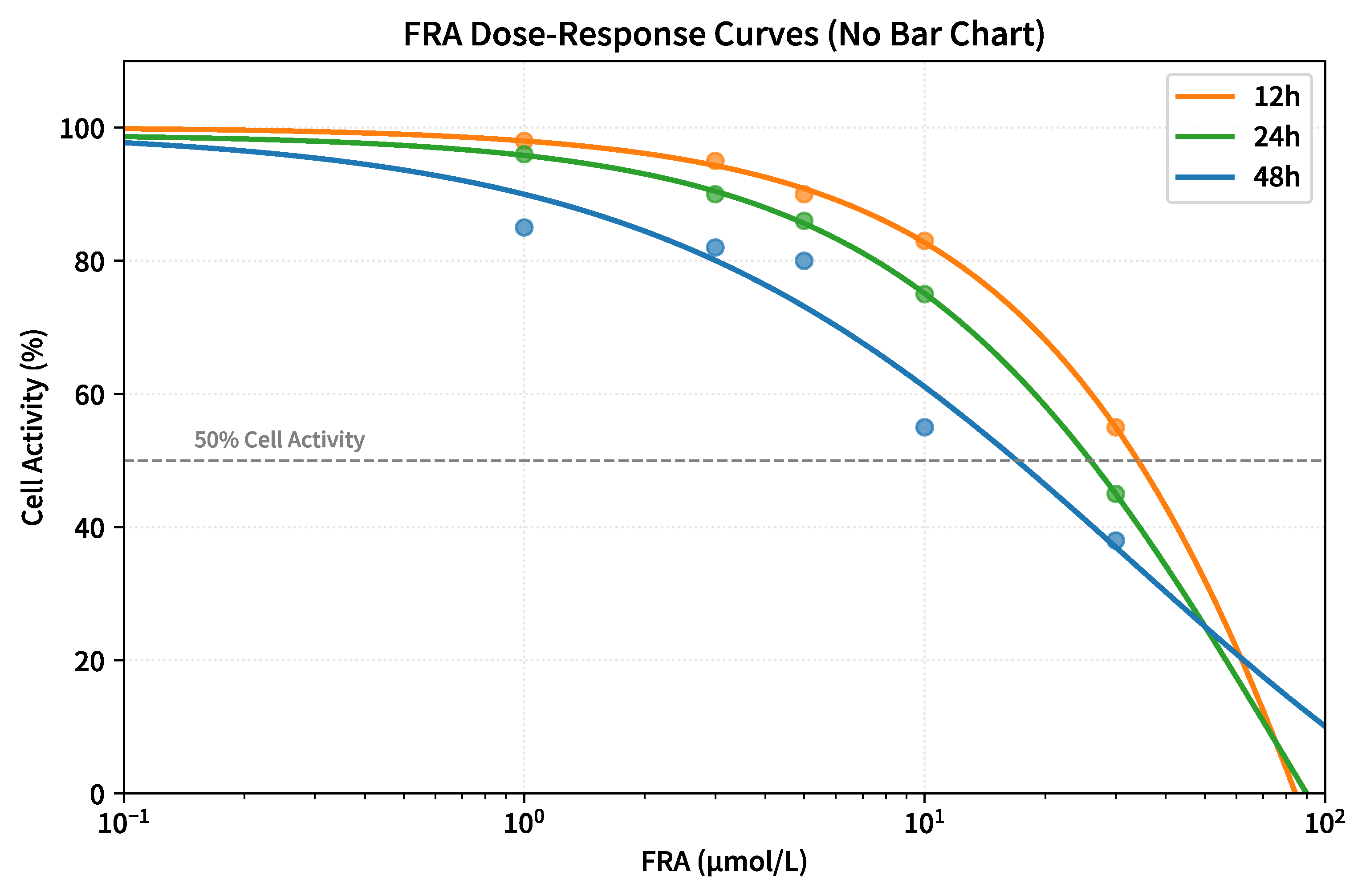

Supplement: Supplementary Figure 2 — Dose-response curves of FRA treatment at different exposure times. Cells were treated with increasing concentrations of FRA (0–30 μmol/L) for 12, 24, and 48 h, and cell viability was assessed using the CCK-8 assay. FRA exhibited both concentration-dependent and time-dependent inhibitory effects on cell viability. The dashed line indicates 50% cell activity, which was used to estimate the approximate half-maximal inhibitory concentration (IC50) values for each treatment duration. Based on these results, 2.5, 5, and 10 μM were selected for subsequent mechanistic experiments to avoid excessive cytotoxicity. [file Image2.tif]
